# Effect of seaweed (*Ecklonia cava* extract) on blood glucose and insulin level on prediabetic patients: A double‐blind randomized controlled trial

**DOI:** 10.1002/fsn3.3133

**Published:** 2022-12-07

**Authors:** Malak Ghazi Almutairi, Khalid Aldubayan, Haneen Molla

**Affiliations:** ^1^ Department of Clinical Nutrition Almethnab General Hospital, Ministry of Health Riyadh Saudi Arabia; ^2^ Community Health Sciences Department, college of Applied Medical Sciences King Saud University Riyadh Saudi Arabia; ^3^ Director of Clinical Nutrition Department, King Khalid University Hospital King Saud University Medical City Riyadh Saudi Arabia

## Abstract

To investigate the effect of polyphenolic‐rich seaweed extract (*Ecklonia cava*) on postprandial blood glucose (PPBG) and postprandial insulin level (PPIL) as well as investigating any associated side effects related to the study intervention in 20 prediabetic patients in Saudi Arabia. The double‐blind, randomized‐controlled trial was conducted from November 2020 to April 2021 in Riyadh, in 20 prediabetic patients with no other health complications. Subjects were given 600 mg of seaweed extract in a single dose for acute effect investigation. PPBG and PPIL were measured immediately at intervals of 30, 60, 90, and 120 min following 75 g of carbohydrate consumption, iAUC and peak concentration were calculated accordingly. Insignificant differences were shown for PPBG levels between study groups at intervals of 30 and 60 min (*p* > .05). However, PPBG results were significantly lower in the intervention group compared to placebo of 90 and 120 min after carbohydrate (75 g) consumption. The mean (SD) of PPBG in the seaweed group at 90 and 120 min was 108.1 (±8.9) and 101.3 (±8.7), respectively, compared to the placebo group at 90 and 120 min with a mean of 122.2 (±16.9) and 112.9 (±12.1), respectively (*p* value at 90 min = 0.032) and (*p* value at 120 min = 0.024). iAUC of PPBG shows no significant differences between the study groups (*p* > .05). There was no significant difference in PPIL between study groups at all study measurements (*p* > .05). Discomfort symptoms were similar between study groups (*p* > .05). This study indicated that a single dose of 600 mg of E. cava extract has a lowering effect on postprandial blood glucose with no associated side effects. Further research should investigate the glycemic modulating effects of marine algal extracts in the long‐term investigation.

## Graphical abstract

To investigate the effect of seaweed extract called Ecklonia cava on postprandial blood glucose (PPBG) and postprandial insulin level (PPIL) as well as investigating any associated side effects using a double‐blind, Randomized‐controlled trial that was conducted from November 2020 to April 2021 in Riyadh, in 20 prediabetic subjects with no other health complications. Subjects were given 600 mg of seaweed extract in a single dose for acute effect investigation.

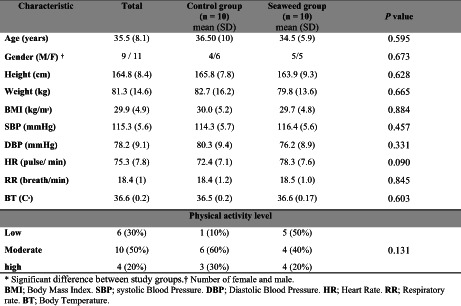

## 1 INTRODUCTION

Type 2 diabetes mellitus (T2DM) affects hundreds of millions of people worldwide, and the prevalence is rapidly increasing in most parts of the world. According to the World Health Organization (WHO) global report on diabetes (World Health Organization, 2019) and the ninth edition of the International Diabetes Federation (IDF) (International Diabetes Federation, 2019), the prevalence of T2DM has increased from 108 million in 1980 to over 463 million in 2019. The prevalence is predicted to reach over 700 million people accounting for 10.9% of the world's adult population in 2045 (International Diabetes Federation, 2019). Saudi Arabia has been ranked the second highest rate of diabetes in the Middle East and seventh highest in the world by WHO (Diabetes Fact Sheet No312, 2018). In 2014, a systematic review reported that the prevalence of T2DM in Saudi Arabia is around 32% of those aged 20–79 years and this trend is estimated to rise to 35.37% in 2030 (Alharbi et al., 2014). T2DM is commonly characterized by either insufficient insulin secretion or insulin resistance (International Diabetes Federation, 2019). In a normal physiological state, the insulin level is constantly released into the bloodstream by pancreatic β‐cells after food ingestion. The postprandial increase in insulin level as well as the increased level of blood glucose inhibits the renal and hepatic secretion of glucagon into circulation and stimulates glucose uptake by various tissues (Kim et al., 2017). Postprandial decrease in insulin secretion leads to less inhibition of glucagon hormone, resulting in higher hepatic and renal glucose production and a reduction in glucose uptake by cells, thus hyperglycemia occurs (DeFronzo et al., 2015). Postprandial hyperglycemia is a common biochemical sign of T2DM and prediabetes. Commonly, prediabetes is a result of impaired fasting glucose (IFG) and/or impaired glucose tolerance (IGT) (International Diabetes Federation, 2019). American Diabetes Association (ADA) has defined prediabetic patients who have the value of oral glucose tolerance test (OGTT) between 140 mg/dl and 199 mg/dl, a level of Ab1c between 5.7% and 6.4%, or fasting blood glucose (FBG) level from 100 mg/dl to 125 mg/d (American Diabetes Association, 2020). Inhibition of α‐amylase and α‐glucosidase enzymes (carbohydrate digestive enzymes) is a suggested strategy for glycemic control, particularly in individuals with prediabetes and T2DM (Tundis et al., 2010). α‐amylase and α‐glucosidase are carbohydrate digestive enzymes that are capable of hydrolyzing α‐glucans (starch and glycogen), oligosaccharides, and disaccharides into simple sugars. These enzymes act on α (Alharbi et al., 2014; World Health Organization, 2019) glycosidic bonds; the bonds that link carbon‐1 of one monosaccharide with carbon‐4 of the other monosaccharide (Berg et al., 2002). Therefore, pharmaceutical industries have developed α‐amylase and α‐glucosidase inhibitors, which are commonly associated with adverse effects such as abdominal discomfort, bloating, and diarrhea (Lorenzati et al., 2010). However, some food components have been suggested to have similar effects with no or fewer side effects (Tundis et al., 2010). Seaweeds, also called marine algae, are considered high‐quality healthy food as it contains diversified bioactive compounds which exhibit various beneficial biological effects (Sharifuddin et al., 2015). Brown seaweed, containing polyphenolic extracts, such as catechins, phlorotannins, and flavonoids which are well‐known as functional foods components (Cofrades et al., 2010), has been proposed to effectively inhibit α‐amylases and α‐glucosidases, therefore improving diabetic‐related response (Sharifuddin et al., 2015). A recent systematic review has shown a desirable effect of brown seaweed on postprandial blood glucose and insulin in healthy individuals (Coe & Ryan, 2016). Also, acute improvement in insulin sensitivity was seen in healthy individuals when taking brown seaweed after consumption of a carbohydrate meal (Paradis et al., 2011). The postprandial hypoglycemic effect of brown seaweed has been demonstrated among individuals with high blood glucose in a 12‐week intervention supplementation by lee and Jeon al in 2015 (Lee & Jeon, 2015). Also, a high dose (2000 mg) of the same seaweed extract intervention yielded nonsignificant results when compared to placebo as illustrated by Murray et al. (2018). Therefore, the present study investigates the effect of brown seaweed called *Ecklonia cava* (E. cava) on postprandial blood glucose (PPBG) and insulin level (IL) in individuals diagnosed with prediabetic patients in Saudi Arabia.

## 2 MATERIALS AND METHODS

### 2.1 Study design

A double‐blind, randomized controlled trial was conducted from November 2020 to April 2021 in Riyadh, Saudi Arabia. The study has been registered at Saudi Clinical Trial Registry (SCTR) by Saudi Food and Drug Authority (SFDA) with registration number—20092302 as well as at ClinicalTrials.gov with ID—NCT 04864860 (available at https://clinicaltrials.gov/ct2/show/NCT04864860). Ethical approval was granted by the Institutional Review Board (IRB) at King Saud University (KSU) (Approval No: E‐19‐4249). Written consent forms were obtained from all participants prior to study participation and they were able to withdraw from the study at any time for any reason. All procedures were conducted according to the Declaration of Helsinki.

### 2.2 Participants

Participants were eligible to participate if they were diagnosed in their medical history as the prediabetic patient aged between 18 and 65 years with fasting plasma glucose (FPG) between 100 and 125 mg dl − 1 based on diagnostic criteria for prediabetic set by American Diabetic Association (Li et al., 2017), and blood pressure within the normal range (systolic blood pressure ≤ 140 mmHg, DBP ≤ 90 mmHg set by WHO) having no other health complications. Participants were excluded if they were taking any treatment with either insulin or antidiabetic drugs or any other natural health products known to impact blood sugar, or polyphenol absorption (e.g., fish oil). Also, they were excluded if they were smokers, pregnant or lactating, or having liver, thyroid, or significant gastrointestinal disorders. Participants were recruited and screened by a research team using flyers, snowballing, and a personal approach at King Khalid University Hospital (KKUH) in Riyadh city in the Kingdom of Saudi Arabia.

### 2.3 Intervention

The intervention capsule is 600 mg of *Ecklonia cava* extract called “Seanol” which contains 13% phlorotannic polyphenol per capsule as stated by the manufacturing company (Seanol inside, 4215 95th St SW Lakewood, WA 98499). Other ingredients are dextrin, magnesium stearate, and silica (in a neglected percentage). This dose was selected to be similar to previous studies that showed no harm or severe adverse effect on participants (Shin et al., 2012; Paradis et al., 2011). The placebo is dextrin (BETA CYCLODEXTRIN, NF) ordered from a pharmaceutical company “MEDISCA”. Dextrin was selected to account for the similar complex carbohydrate content of the intervention supplement. Placebo was encapsulated in vegetable cellulose capsules that are identical in size and color to the intervention capsules. Intervention and placebo were encapsulated in the laboratory at the pharmacy department at King Saud University under the supervision of a professor from the pharmacy faculty.

### 2.4 Randomization and masking

Study randomizations were administered by encoding participants with numbers, and supplementation with letters (A, B). Computer‐generating randomization was used to randomly select participants from eligible subjects. The sequence that the participants received their supplements as either study intervention (E. cava) or placebo was determined using the same procedure. The identity of the supplements was only known to an investigator who was not involved in data collection and analysis. Participants and investigators who conducted recruitment, data collection, and data analysis were blinded to which supplement is consumed on each occasion until data analysis was completed.

### 2.5 Study procedure

Study recruitment was managed first by using phone interviews with interested participants. Participants who meet inclusion criteria were screened at the clinics of the Clinical Trial Unit at KKUH to ensure that fasting blood glucose is in the range between 100 and 125 mg dl−1, and blood pressure is within the normal range (SBP ≤ 140 mmHg, diastolic blood pressure ≤ 90 mmHg). Also, participants who are having any health issues listed in the exclusion criteria were excluded. Then, recruited participants were randomly selected, then they were randomly assigned to either placebo or *E. cava* group. Then, they were invited to the testing occasions with specific dates and times (only one testing occasion for each participant which lasted for 3 h).

All participants in both groups were informed to fast for 10 h (except water) with minimum exercise as possible before being admitted to the testing room in order to eliminate any expected confounders. Food was not provided a day prior to the testing occasion; however, participants were informed to have a standardized evening meal between 7 and 9 p.m. Anthropometric data weight, height, and demographic information, age and sex, were collected. Blood pressure has been taken by a registered nurse during an experimental visit after resting for 5 min in the clinic. Participants were asked about their history of chronic diseases and each case was confirmed by the medical records. Participants' physical activity has been recorded and categorized as low, moderate, and high. The 24‐h dietary recall was collected during testing occasions for dietary analysis using FoodWorks software, which is designed for Australia and New Zealand.

**FIGURE 1:**
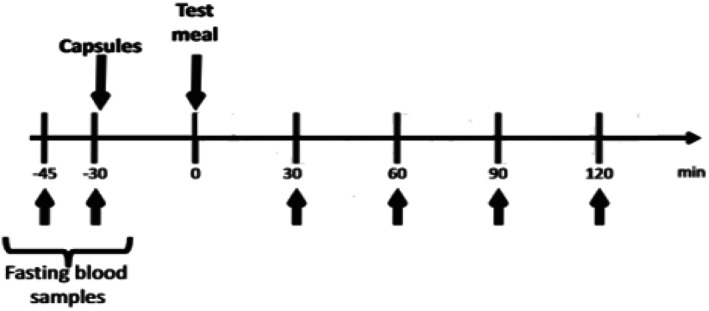
Timeline for blood samples collection pre‐ and post‐test meal (75‐g carbohydrates).

As shown in Figure 1, on a testing occasion two fasting (> 10 h) blood samples were taken at −45 and −35 min prior to carbohydrate consumption to determine average FBG and insulin levels. At the time of −30 min, either a study test (E. cava extract) or placebo capsules were administered to participants based on the randomization order. Then, at time 0, participants consumed 110 gm of white bread (four slices), which contain approximately 75 g of carbohydrates. Blood samples were collected in accordance with the standard oral glucose tolerance test protocol (McDonald et al., 1965) for the following 2 h of test meal consumption at intervals of 30, 60, 90, and 120 min for blood glucose and insulin level, respectively.

On the testing occasions, the PPBG level was measured immediately after taking blood samples using a glucometer. However, samples collected for insulin levels were measured at the KKUH laboratory, then results were obtained.

In order to assess the side effects of the study intervention, participants were asked to indicate whether side effects are absent, mild, moderate or severe by giving scores of 0, 1, 2, and 3, respectively. The side effect questionnaire, which was adapted from the intolerance symptoms questionnaire used by Paradis et al (Paradis et al., 2011), was required to be completed within 24 h of follow‐up by phone after intervention or placebo ingestion. The side effect questionnaire included headache, energy levels, appetite, gastrointestinal symptoms, unusual pain or sensations, cardiac palpitations, balance disorders, and depression/anxiety.

### 2.6 Outcome measurement

Plasma glucose concentration was determined immediately by blood finger‐prick sample following standard procedure using Accu‐Chek Instant (Roche Diabetes Care, Inc., Indianapolis, Indiana), whereas plasma insulin was measured at the laboratory department at KKUH using enzyme‐linked immunosorbent assay (ELISA).

### 2.7 Sample size

Power calculation and sample size determination were performed based on data reported in similar studies (Murray et al., 2018; Paradis et al., 2011), which shows that 12 participants are required in each group. These numbers of individuals were required to detect differences of 38 units in BG and 2500 units in plasma insulin (incremental area under the curve (iAUC) at 0.05 significance level with 80% power of the study). For the purpose of allowing 10% participants withdraw, the target number of recruitments was 30 individuals.

### 2.8 Statistical analysis

After data collection, BMI was calculated using the equation weight in kg/height in m^2^. Data were assessed for normality using the Shapiro–Wilk test. Therefore, based on normality tests, parametric tests were used. Mean (standard deviation) was reported for normally distributed data. An independent *t*‐test was used to determin the difference between the two groups for both PPBG and postprandial IL at a significant level of *p* < .05. Mann–Whitney test was used to determine differences for symptoms of intolerance between groups. Incremental area under the curve (iAUC), time to peak, and peak blood concentration assessment were used to assess postprandial responses for plasma glucose and insulin level. Statistical analysis was performed using the Statistical Package for Social Sciences (SPSS) version 20 (SPSS Inc., Chicago, IL).

## 3 RESULTS

### 3.1 Demographics and baseline characteristics

All participants were randomized on a computer‐generated protocol by the Investigational Drug & Research Unit (IDRU) into two groups: First group as control and second one with seaweed supplementation group. A total of 30 participants were recruited from November 2020 to April 2021. However, 10 participants withdrew from the study after enrollment due to participants' lack of time (three participants due to night work duty and seven due to restrictions of COVID‐19). Only 20 participants (nine men and 11 women) have completed the study protocol. The baseline characteristics of study subjects are presented in Table 1. Study participants aged from 21 to 60 years with a mean of 35.3 ± 8.1 years and BMI ranged from 21.5 to 38.4 kg/m^2^ with a mean of 29.9 ± 4.9. Gender was approximately equally distributed among study groups. All recruited subjects in two study groups were homogenous having normal blood pressure, heart rate, respiratory rate, and body temperature as depicted in Table 1. Most study participants have moderate physical activity on the basis of weekly exercise duration (at least 150 min of aerobic activity). Dietary recall has demonstrated insignificant differences in content of energy intake, carbohydrate, protein, and fat between study groups as shown in Table 2.

**TABLE 1:** Study sample characteristics and physical activity level at baseline

Characteristics	Total	Control group (*n* = 10) mean (SD)	Seaweed group (*n* = 10) mean (SD)	*p* value
Age (years)	35.5 (8.1)	36.50 (10)	34.5 (5.9)	.595
Gender (M/F)^b^	9 / 11	4/6	5/5	.673
Height (cm)	164.8 (8.4)	165.8 (7.8)	163.9 (9.3)	.628
Weight (kg)	81.3 (14.6)	82.7 (16.2)	79.8 (13.6)	.665
BMI (kg/m^2^)	29.9 (4.9)	30.0 (5.2)	29.7 (4.8)	.884
SBP (mmHg)	115.3 (5.6)	114.3 (5.7)	116.4 (5.6)	.457
DBP (mmHg)	78.2 (9.1)	80.3 (9.4)	76.2 (8.9)	.331
HR (pulse/ min)	75.3 (7.8)	72.4 (7.1)	78.3 (7.6)	.090
RR (breath/min)	18.4 (1)	18.4 (1.2)	18.5 (1.0)	.845
BT (C^0^)	36.6 (0.2)	36.5 (0.2)	36.6 (0.17)	.603
Physical activity level				
Low	6 (30%)	1 (10%)	5 (50%)	
Moderate	10 (50%)	6 (60%)	4 (40%)	.131
High	4 (20%)	3 (30%)	4 (20%)	

*Note*: Significant difference between study groups (*p* > 0.05).

Abbreviations: BMI, Body Mass Index; BT, Body Temperature; DBP, Diastolic Blood Pressure; HR, Heart Rate; RR, Respiratory Rate; SBP, Systolic Blood Pressure.

^b^ Number of female and male.

**TABLE 2:** Difference of macronutrients intake between control and seaweed groups using 24‐h recall

Groups	Control group (*n* = 10) mean (SD)	Seaweed group (*n* = 10) mean (SD)	*p* value
Carbohydrates (g)	190.1 (21.9)	184.6 (18.7)	.558
Protein (g)	112.1 (18.1)	111.8 (20.1))	.978
Fat (g)	37.5 (4.7)	36.9 (4.9)	.790
Energy (calorie)	1596.6 (200.4)	1571.8 (208.4)	.790

*Note*: Significant difference between study (*p* > .05).

### 3.2 Postprandial blood glucose

**FIGURE 2:**
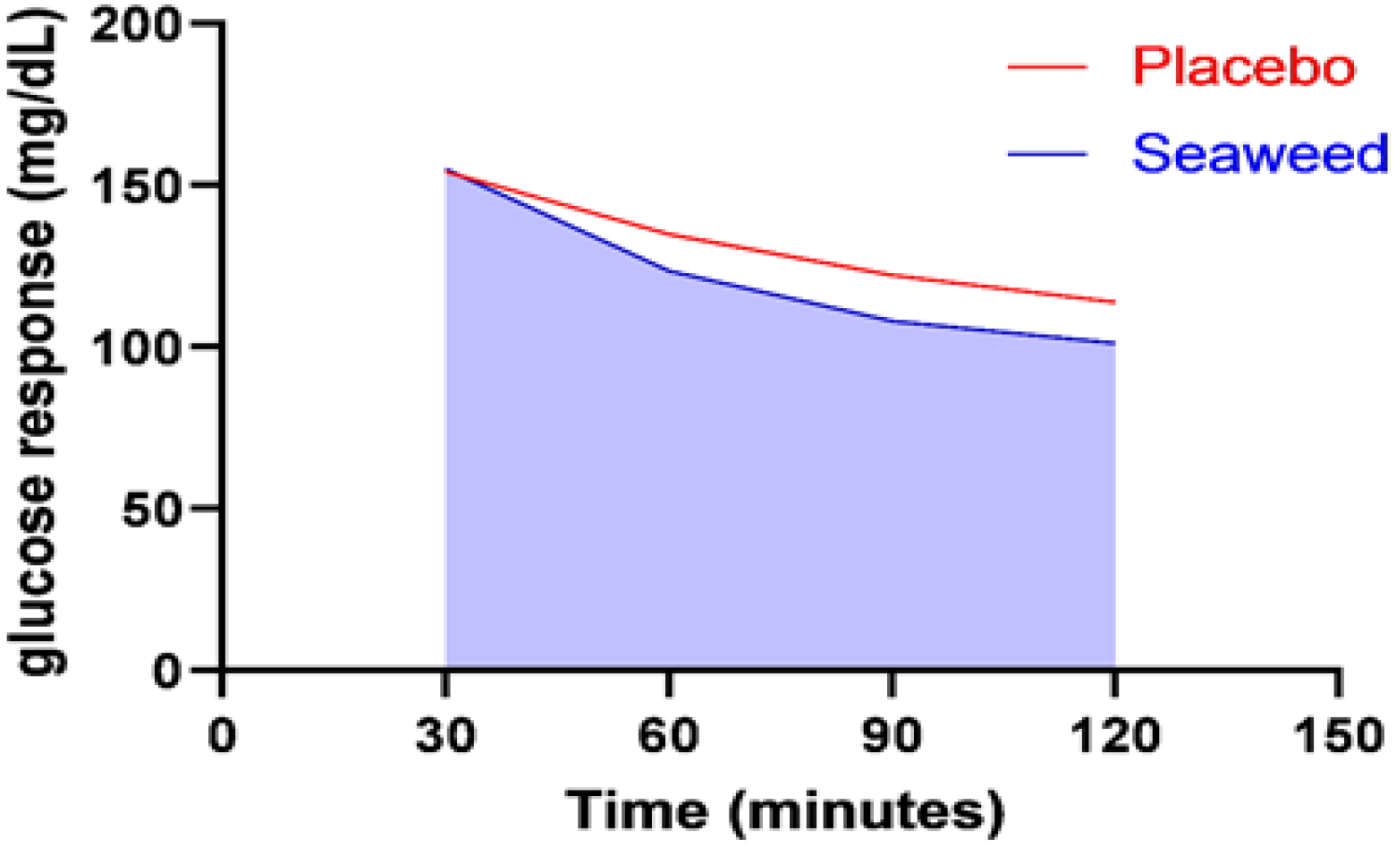
iAUC postprandial blood glucose.

All participants' FBG levels were checked before the consumption of carbohydrate meal with means of 108.4 ± 6.7 mg/dl. Statistical tests showed no significant differences in the FBG level between study groups prior to ingestion of study treatments (mean (SD): 109.5 (±8.2) and 107.2 (±5.0) for seaweed and placebo, respectively, *p* value = .449). Also, insignificant differences were shown for PPBG level between both groups at intervals of 30 and 60 min. However, PPBG resulted in significant differences between study groups at intervals of 90 and 120 min after carbohydrate (75 g) consumption. The mean (SD) of PPBG in the seaweed group at 90 and 120 min was 108.1 (±8.9) and 101.3 (±8.7), respectively, compared to placebo group at 90 and 120 min (122.2 (±16.9) and 112.9 (±12.1), respectively, (*p* value at 90 min = .032) and (*p* value at 120 min = .024)). iAUC of PPBG shows no significant differences between the study groups as illustrated in Figure 2. Also, there are no significant differences in terms of PPBG peak concentration as shown in Table 3.

**TABLE 3:** Fasting and postprandial blood glucose and plasma insulin measures, for placebo and seaweed extracts group

Outcome	Total mean (SD)	Placebo mean (SD)	Seaweed mean (SD)	*p* value
Fasting blood glucose (mg/dl)				
Fasting (mg/dl)	108.4 (6.7)	107.2 (5.0)	109.5 (8.2)	.449
Postprandial blood glucose (mg/dl)				
Postprandial blood glucose (mg/dl) at 30 min	154.6 (22.1)	154.2 (23.2)	155.0 (22.3)	.938
Postprandial blood glucose (mg/dl) at 60 min	129.3 (17.4)	134.9 (17.1)	123.6 (16.8)	.154
Postprandial blood glucose (mg/dll) at 90 min	115.2 (15.0)	122.2 (16.9)	108.1 (8.9)	.032*
Postprandial blood glucose (mg/dl) at 120 min	107.1 (11.8)	112.9 (12.1)	101.3 (8.7)	.024*
iAUC (mg/dl.min)	11,265	11,735 (2035.2)	10,796 (1711.2)	.278
Peak concentration (mg/dl.min)	155.0	154.2	155.0	.875
Fasting blood insulin (mU/L)				
Fasting (mU/L)	12.97 (5.0)	14.15 (4.5)	11.78 (5.5)	.306
Postprandial blood insulin (mU/L)				
Postprandial blood insulin (mg/dl) at 30 min	68.2 (35.5)	78.4 (39.8)	58.1 (29.1)	.207
Postprandial blood insulin (mU/L) at 60 min	64.4 (47.3)	63.8 (24.6)	64.8 (64.1)	.963
Postprandial blood insulin (mU/L) at 90 min	44.7 (39.4)	46.4 (27.8)	43.1 (49.9)	.860
Postprandial blood insulin (mU/L) at 120 min	40.1 (49.3)	31.8 (15.8)	48.4 (15.8)	.475
iAUC (mU/L.min)	4900 (5037)	4955 (3222)	6227 (6647)	.592
Peak concentration (mU/L.min)	68.3	78.4	65.0	.263

Significant difference between study groups (*p* > .05).

Abbreviations: iAUC, incremental Area Under the Curve.

### 3.3 Postprandial insulin level

**FIGURE 3:**
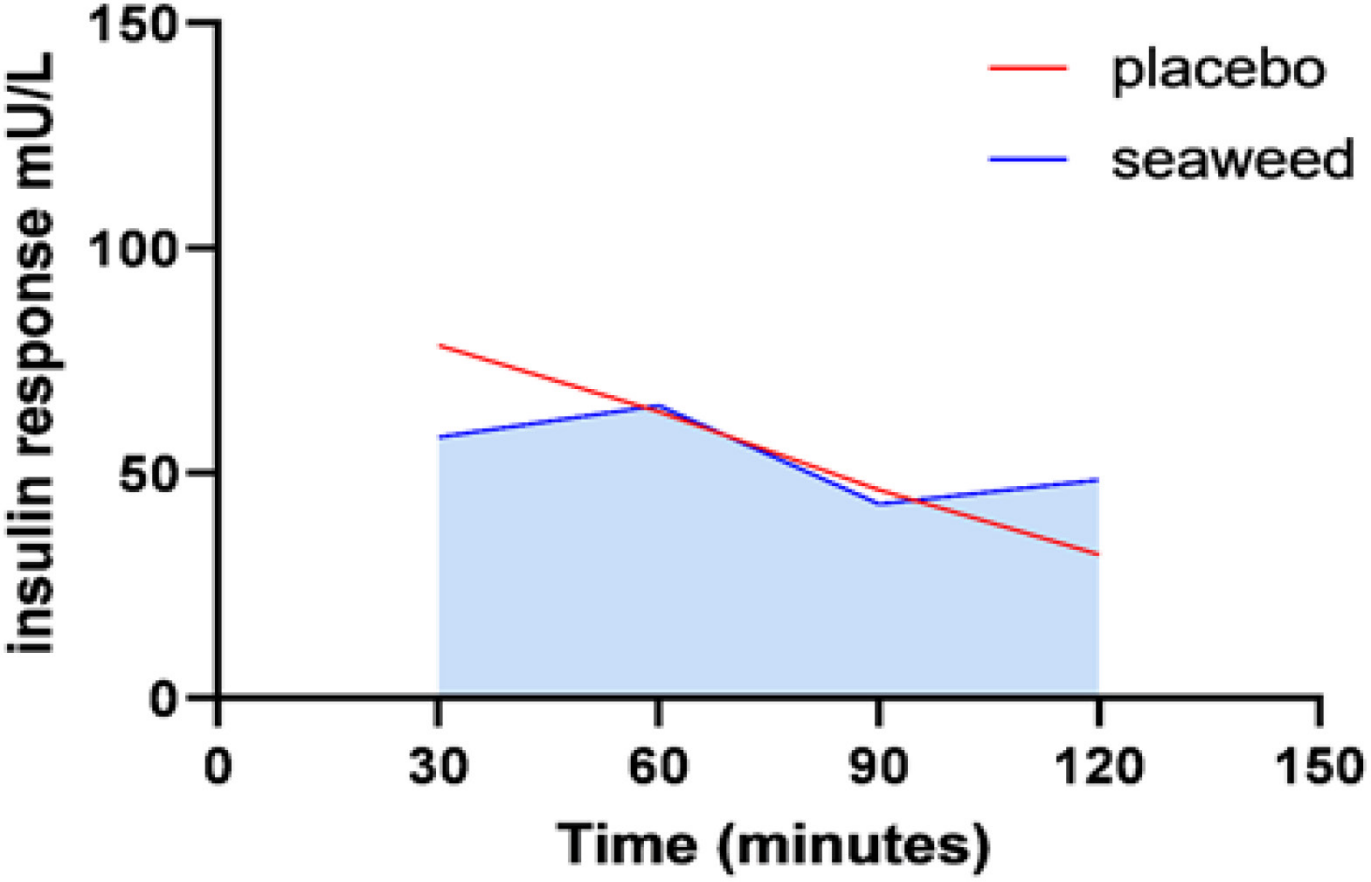
iAUC postprandial insulin level.

Prior to treatment ingestion, no significant differences were observed for fasting insulin levels in seaweed and placebo groups; 14.15 ± 4.5 vs 11.78 ± 5.5 mU/L, respectively. Postprandial IL was not significant after all the different interval measurements between the seaweed extract group compared to the placebo group. iAUC of PPIL is lower in the placebo group compared to the seaweed extract group; however, no significant differences were observed (*p* value > .05) (Table 3). As shown in Figure 3, mean postprandial IL in the control group continued to reduce from 78.4 (±39.8) to 40.1 (±49.3), while in the seaweed group the mean postprandial IL fluctuated during the time intervals of postprandial insulin measurements. Also, postprandial IL peak concentrations have no significant difference among seaweed groups compared to placebo.

### 3.4 Side effect

Intolerance symptoms between the treatment groups were similar. Only one participant in the placebo group reported intolerance symptoms which are headache, fatigue, and lack of energy and were rated as moderate (2 on the arbitrary scale of 0 to 3). Thus, there was no significant difference between seaweed extracts and placebo groups in intensity and/or frequency of discomfort symptoms.

## 4 DISCUSSION

In this double‐blinded, randomized control trial, the effect of brown seaweed (E. cava) extracts on PPBG and insulin level in prediabetic subjects was investigated. In our study, most of the participants were overweight and calorie intake was normal as per RDA during recent 24‐h diet recall. Reduction of PPBG level among seaweed supplemented group has been found at the intervals of 90 and 120 min, findings of this study were in agreement with Lee and Jeon et al, 2015 and Shin et al, 2008 (Shin et al., 2012; Lee & Jeon, 2015). A previous study by Lee and Jeon has shown improvement and reduction of PPBG response with meals of cooked rice after 12‐week intervention in subjects with a high baseline fasting blood glucose level (100–180 mg/dl) (Lee & Jeon, 2015). Our findings also were consistent even on acute response after a single dose of 600 mg of E. cava supplementation. However, a study by Paradis et al (2014) showed that using 25 mg of *Fucus vesiculosus* and *Ascophyllum nodosum* extracts did not show significant results in regard to PPBG and insulin level among healthy subjects with normal weight (Paradis et al., 2011). Also, a high dose (2000 mg) of the same seaweed extracts intervention yielded nonsignificant results when compared to a placebo as illustrated by Murray et al, (2018) (Murray et al., 2018). This inconsistency of findings could be a result of different seaweed extracts they used in their trials. However, the content of polyphenol was 13% with 85% of powdered dextrin in our study which is similar to the content used in the study of Pardis et al, while in contrast, it is very low compared to Lee and Jeon's study (40%) (Lee & Jeon, 2015). These findings suggest that different types of polyphenols with different sources might be acting differently in the body (Malik & Mukherjee, 2014; Tsao, 2010), which may explain why a reduction in postprandial blood glucose was observed following the intervention in our study but not Paradis et al. (2011) and Murray et al (2018) (Murray et al., 2018; Paradis et al., 2011). Currently, E. cava is considered the most extensively studied source of marine polyphenols for antihyperglycemic effects and it has shown the most promising results in this area in animal models (Kang et al., 2010; Kang et al., 2013; Lee et al., 2012; Tsao, 2010). Also, the source and amount of carbohydrates may influence the magnitude of the change in PPBG since the result of acute effect on PPBG level did not highly decrease the meal (white bread). In our study, glycemic index was different when compared to cooked rice in Lee and Jeon's study (Lee & Jeon, 2015). Importantly, the efficacy of seaweed extract intervention may only be induced in populations with glycemic dysregulation as found in our study and Lee and Loen's study which opposed healthy populations that were involved in Paradis et al and Murray et al (Murray et al., 2018).

In this study, postprandial IL concentration was not significantly changed after supplementation of brown seaweed extract to prediabetic subjects as compared with the control group. This finding agrees with most previous clinical trials conducted in humans. However, in Lee and Joen's study, a significant within‐group, but not between‐groups, reduction was seen in the E. cava group after 12 weeks of intervention (Lee & Jeon, 2015). Similarly, fasting insulin level following brown seaweed supplementation for a period of time in different studies (Lee & Jeon, 2015; Paradis et al., 2011) showed conflicting results. After 3 months of supplementation, fasting insulin increased with a low dose of polyphenols (25 mg/d) (Hernandez‐Corona et al., 2014), whereas it decreased with a high dose (690 mg/d) (Lee and Jeon, 2015) (Hernández‐Corona et al., 2014; Lee & Jeon, 2015). In Hernandez‐Corona et al (2014), insufficient therapeutic dose level may be the reason behind reduced efficacy in fasting insulin level, taking into consideration that a similar increase in fasting insulin level was observed in the control group as well (Hernández‐Corona et al., 2014). In comparison, the source of high‐dose supplementation was a dieckol‐rich E. cava extract (46% polyphenols) where antidiabetic effects, including reduction of fasting blood insulin levels, were evident in multiple animal studies (Kang et al., 2010; Kang et al., 2013;Lee et al., 2012; Park et al., 2012). This indicates that alteration in insulin threshold may need long‐term supplementation in addition to the specified type and therapeutic dose of seaweed extracts used. Therefore, further research is required to investigate the effective dose as well as determine the consistency of the seaweed extract effect.

The hypothesis of this study was based on in vivo and in vitro evidence that suggests the antidiabetic effect of polyphenolic‐brown seaweeds including E. cava extracts (Lee et al., 2012; Park et al., 2012). The main suggested mechanism of antidiabetic effects of brown seaweed‐derived polyphenols on postprandial glycemic response is by inhibiting the carbohydrate enzymes, namely, a‐amylase and a‐glucosidase enzymes (Heo et al., 2009; Lee et al., 2009; Zhang et al., 2007). This is clearly demonstrated in the study of Lee et al, where dieckol isolated from E. cava exhibited a‐glucosidase and a‐amylase inhibitory activities and also reduced postprandial hyperglycemia in streptozotocin‐induced diabetic mice (Lee et al., 2012). In addition, Shin et al. found that the phlorotannins extracted from E. cava exhibited strong antioxidant potential which helps to reduce the oxidative stress‐induced complications of diabetes. However, when these investigations were based on humans, few clinical studies failed to yield clear and consistent results.

Moreover, side effects in our study were similar between study groups, which consisted of results of similar studies (Lee & Jeon, 2015;Murray et al., 2018; Paradis et al., 2011). The maximum reported dose in human studies was up to 1500 mg taken daily for 12 weeks, showing no adverse effect on study subjects (Lee & Jeon, 2015). The safety of *Ecklonia cava* consumption in humans was assessed by the Food and Drug Authority (FDA) in the USA and European Food Safety Authority (EFSA) and both authorities granted safety approval as a new dietary ingredient for supplementation (Retrieved from, 2022; EFSA Panel on Dietetic Products, Nutrition and Allergies et al., 2017). FDA and EFSA reports have shown that E. cava extracts are not mutagenic or cytotoxic to the cellular system. EFSA recommended the use of food supplements at a maximum daily intake level of 263 mg/day for adults (EFSA Panel on Dietetic Products, Nutrition and Allergies et al., 2017). Whilst the FDA did not set an upper limit for the human consumption of E. cava depending on the history of use in food and supplementation as well as previous preclinical and clinical studies (Retrieved from, 2022). This suggests the safe use of seaweed extract as a potential antidiabetic use. However, the threshold of therapeutic dose along with upper limits needs more investigation.

The current study is the first study investigating the effect of seaweed extract in patients diagnosed in their medical history with prediabetes but not yet using antidiabetic or other medications. Investigation in subjects with dysregulated glycemia was recommended by previous studies that failed to find out the antidiabetic effect of seaweed in healthy subjects (Murray et al., 2018; Paradis et al., 2011). On the other hand, the outstanding limitations of this study include a high withdrawal rate (around 30%). This study has been conducted during the spread of COVID‐19 pandemic and during periods of public restrictions which may impact participants' compliance. The study methodology was designed to investigate the acute effects of glycemic response which could be a limitation due to the effects of physical activity and dietary intake on PPBG and IL prior to conducting the trial. However, considerations were taken as participants were instructed to reduce their physical activity and consume a typical diet a day before their testing occasion. Also, studying acute result design can be promising for future research.

## 5 CONCLUSION

In conclusion, this study indicated that a single dose of up to 600 mg of a polyphenol‐rich *Ecklonia cava* extract has an additional lowering acute effect over that of 600 g of dextrin on postprandial blood glucose, but no acute effect plasma insulin in prediabetic adults. Also, algae extracts have no potential side effect, suggesting potential use as an antihyperglycemic lowering agent. Further research should investigate the glycemic modulating effects of polyphenol‐rich marine algal extracts in the long‐term investigation, as this is where effects have been identified to date. Based on these results, E. cava extracts could be a promising antidiabetic agent or pharmaceutical resource that may help to improve the quality of life for diabetes‐related populations.

## CONFLICT OF INTEREST

All authors declare that they have no competing interests.

## Data Availability

The data that support the findings of this study are available from the corresponding author upon reasonable request.
